# Does Finnish hospital staff job satisfaction vary across occupational groups?

**DOI:** 10.1186/1472-6963-13-376

**Published:** 2013-10-02

**Authors:** Tarja Kvist, Raija Mäntynen, Katri Vehviläinen-Julkunen

**Affiliations:** 1 Department of Nursing Science, University of Eastern Finland, P.O. BOX 1627, FI-70211 Kuopio, Finland; 2 Kuopio University Hospital, P.O. BOX 1777, FI-70211 Kuopio, Finland

**Keywords:** Job satisfaction, Hospital staff, Physician, Nurse, Web-based survey

## Abstract

**Background:**

Job satisfaction of staff is an essential outcome variable in research when describing the work environment of successful hospitals. Numerous studies have evaluated the topic, but few previous studies have assessed the job satisfaction of all staff in hospital settings. It is important to discover if there are any unsatisfied groups of people working in hospitals, the aspects they are unsatisfied with and why. The aim of this study was to evaluate job satisfaction of all staff working at a Finnish university hospital, identify differences in job satisfaction between staff groups, and explore the relationship between their self-evaluated quality of work and job satisfaction.

**Methods:**

Data were collected from 1424 employees of the hospital using the web-based Kuopio University Job Satisfaction Scale survey instrument in autumn 2010. The research data were analysed by using SPSS 19.0 for Windows. Frequency and percentage distributions, as well as mean values, were used to describe the data. A non-parametric test (Kruskal–Wallis test) was used to determine the significance of differences in scores between different groups of staff members and between quality evaluations.

**Results:**

The overall job satisfaction of the employees was good. They rated both motivating factors of their work and work welfare as excellent. The areas causing most dissatisfaction were work demands and participation in decision making. Physicians formed the most satisfied group, nurses and maintenance staff were the least satisfied, and office and administrative staff were fairly satisfied. Staff who rated the quality of work in their units as high usually also considered their job satisfaction to be excellent.

**Conclusions:**

Every staff member has an influence on job satisfaction in her/his unit. A culture of participation should be developed and maintained in the units and the whole hospital to ensure that all staff feel they play important roles in the hospital. A university hospital is a complex, continuously changing work environment. Managers of the hospital should continuously evaluate job satisfaction and quickly react to the results gained.

## Background

Job satisfaction of staff is an essential outcome variable in research describing the work environment of successful hospitals, and hence it has been evaluated in numerous studies. Several studies have focused on the job satisfaction of nursing staff, and provided evidence (inter alia) that it is a particularly important success factor and quality outcome of Magnet hospitals. The Magnet hospital concept and program was developed in the United States over 20 years ago. The American Nurses Credentialing Center (ANCC) grants the Magnet designation to hospitals,that have proved e.g. an excellent patient safety record, patient satisfaction, job satisfaction, transformational leadership and patient outcomes. Since its introduction, the Magnet designation has been extended worldwide [1-4]. Job satisfaction among other healthcare personnel groups, especially physicians, has also been studied [5-12]. However, few previous studies have assessed the job satisfaction of all staff in the hospitals [5,11]. Therefore, to address this gap in knowledge and identify areas where improvements are required, we studied the job satisfaction of all staff in one university hospital using a survey instrument and scales developed as a part of a project conducted by the Department of Nursing Science of the University of Eastern Finland and Kuopio University Hospital (KUH) entitled “Attractive and Safe Hospital (At Safe)”.

Job satisfaction can be defined as the extent to which employees like their jobs [10,11,13]. It is an emotional state of individuals that is enhanced by achieving desired results at work [14] and the feeling of belonging to an efficiently functioning work community [15-19]. Job satisfaction is also influenced by working conditions [19,20], internal factors in the workplace, as well as employee attitudes and behaviour [10,21].

Interactions between nurses and nursing leaders also play important roles in nurses’ job satisfaction [13,22,23]. Several studies have found evidence that nursing staff wish that their leaders would: give them more feedback, support and encouragement; understand them better; treat them equally; and set goals together [20,24-28]. Thus, nursing leaders’ leadership style and strengthening of human resources, have a notable impact on job satisfaction among nurses. Similarly, Aasland *et al*. [7] found evidence that Norwegian physicians’ job satisfaction was connected with organisational factors, leadership and economic systems of healthcare.

Job satisfaction is positively correlated with the quality of care, which is thus highest in units where job satisfaction among the staff is high. Hospitals that score highly in job satisfaction measurements also reportedly score highly in quality of care and other desirable outcomes [5,9,11,15]. In addition, a previous Finnish study [29] found that nursing staff members’ and physicians’ perceptions of the quality of care were explained by factors related to job satisfaction as measured in this study. The factors were work (amount, content, development, and being part of organization) and values prevalent in the work environment and organization (eg. the possibility to decide about one’s work).

It is important to avoid excessive generalisations, as there may be substantial variations in job satisfaction among hospitals within the same country, between hospitals in different countries, and among different groups of employees within the same hospital. For example, several studies have indicated that nursing staff working in Magnet hospitals or hospitals pursuing Magnet hospital status are most satisfied with their jobs [2,3]. Physicians’ job satisfaction has been found to be high in Norway [7], good in both Germany [11] and the USA [6,9], and higher than that of nurses and other people working in hospitals in Taiwan [5]. In contrast, Jönsson [10] found that nurses were more satisfied with their psychosocial working environment than physicians in Sweden and identified factors that significantly explained job satisfaction among both groups, including control, social support, qualitative perceptions of role conflicts and clarity of roles. Etchegaray *et al*. [8] reported that nurses and physicians in a Texan hospital gave similar weightings to the factors considered when rating their job satisfaction, and thus conceptualized job satisfaction in a similar manner. In contrast, other researchers (e.g. Chang *et al*. [5], Jönsson [10]) have found that the weightings physicians and nurses apply when evaluating job satisfaction (and thus the power of the factors for predicting their job satisfaction) strongly differ.

In conclusion, numerous previous studies [5-7,9-11,20,23-27] have found that job satisfaction among hospital staff is generally good. Most of these studies have focused on the job satisfaction of nursing staff [4,20,23-27], whereas fewer have examined the job satisfaction of physicians [6,7,9,11], and only one study has considered the job satisfaction of all staff [5]. These previous studies have reported that physicians are very satisfied or satisfied in various countries [6,7,9,11] and generally more satisfied than nurses or other staff groups [5]. However, it is important to identify whether there are any unsatisfied groups of people working in hospitals, the aspects they are unsatisfied with and why in order to provide information to health care leaders that can be used to improve staff job satisfaction.

The objectives of this study were to evaluate the job satisfaction of all staff working at one Finnish university hospital, identify differences in job satisfaction between staff groups and explore the relationship between their self-evaluated quality of work and job satisfaction.

## Methods

### Study design, participants and data collection

Data were collected by a cross-sectional survey of all staff in one university hospital in the autumn of 2010, using the Kuopio University Hospital Job Satisfaction Scale (KUHJSS) instrument, developed as part of the At Safe project [23]. The university hospital has 770 beds. A web-based questionnaire was sent by e-mail to all staff (N = 4357) of the hospital. In total, 1494 members of the staff responded to the questionnaire, but 70 respondents did not provide their job title. Therefore, data from only 1424 respondents were included in the analysis (response rate, 33%). The response rates varied by group: 779/2641 (33%) nurses, 147/619 (24%) physicians, 65/210 (31%) research staff, 237/606 (39%) maintenance staff and 196/281 (70%) office and administrative staff answered the survey. No reminders were sent. Fifty five percent of the respondents (n = 1424) were nursing staff, 10% were physicians, 4% were research staff, 17% were maintenance staff and 14% were office and administrative staff.

### Instrument and its reliability

The Kuopio University Hospital Job Satisfaction Scale (KUHJSS) instrument was originally developed, based on a literature review, for assessing the job satisfaction of nursing staff. Its validity was evaluated by experts, a pilot test with five nurses, and a preliminary test with 503 nurses in 2007. In 2008, it was tested on a much larger scale by surveying the entire nursing staff of four research hospitals (N = 5778) in the Northern Savo Hospital District. Exploratory factor analysis revealed seven factors with modest internal consistency (0.641-0.916) [23].

Based on the results of the above mentioned exploratory factor analysis the KUHJSS used in the present study (Additional file 1) included 37 items divided into seven subscales. The subscales (number of items, an example of the items and Cronbach’s α) were as follows: 1. Leadership (7 items, e.g. *My manager*/*director is genuinely interested in the well*-*being of the staff*, α = 0.914); 2. Requiring factors of the work (8 items, e.g. *My work load is appropriate*, α = 0.802); 3. Motivating factors of the work (6 items, e.g. *My work is interesting*, α = 0,772); 4. Working environment (4 items, e.g. *My work unit is safe and secure*, α = 0.794); 5. Working welfare (4 items, e.g. *I look after my personal well*-*being*, α = 0.641). 6. Participation in decision-making (4 items, e.g. *I have opportunities to make independent decisions in my work*, α = 0.741); 7. Sense of community (4 items, e.g. *I trust the expertise of my colleagues*, α = 0.732). Cronbach’s α for the entire scale was 0.930. Mean scores of responses, ranging from 1 = *strongly disagree* to 5 = *strongly agree*, were computed for all items in each subscale and an overall scale score was computed from the mean scores of the subscales [23].

The KUHJSS also includes 10 demographic variables: hospital, gender, age, working unit, working division, work position, working experience (length of time) in current unit, overall work experience (length of time), type of employment and working hours. In the present study, we did not consider the hospital (only one hospital), or record the working unit and working division (because of anonymity). However, the self-evaluated quality of work in the working unit was asked as a background variable.

### Data analysis

The research data were analysed using SPSS 19.0 for Windows. Frequency and percentage distributions, as well as mean values, were used to describe the data. First, the demographic variables were calculated and then divided into four age classes (≤ 30 years, 31–40 years, 41–50 years and ≥51 years) and five classes of experience in the current work unit and total experience (≤ 1 year, 2–5 years, 6–10 years, 11–20 years and ≥ 21 years). The staff evaluated the quality of work in their working unit using Finnish school grades, which ranged from 4 (worst) to 10 (best), and then, were classified into three groups in based on their ratings: group 1 (poor quality) = 4–6, 2 (moderate) = 7–8 and 3 (excellent) = 9–10.

Normal distributions of the mean scores obtained for the seven subscales were tested with the Kolmogorov–Smirnov test and histograms. Mostly, they did not meet normal distribution criteria for applying parametric tests. Therefore, a non-parametric test (Kruskal–Wallis test) was used to identify any significant differences in scores between different groups of staff members.

### Ethical considerations

This study was approved by the Research Ethical Committee of the Northern Savo Hospital District (Permission number 46/2007). The hospital also issued formal research permission from the chief executive medical director, chief nursing officer and personnel manager. The staff members were informed about the study by the researchers and the leaders of the hospital. The questionnaires included the researchers’ contact details and research information. Participation in the study was voluntary and the staff responded to the questionnaire anonymously. No individual respondent could be identified from the raw data or from the results of the study.

## Results

### Staff demographics

Most of the respondents were women (86%). The respondents’ mean age was 45 (range 20–65) years. The mean ages of the different occupational groups were as follows: physicians 46 (range 28–63) years, research staff 45 (range 23–63) years, maintenance staff 47 (range 21–65) years, office and administrative staff 48 (range 23–63) years and nursing staff 44 (range 20–63) years. The mean length of work experience in the current work unit for the entire staff was 11 (range 0–41) years and total work experience was 19 (range 0–47) years. The mean value of the self-evaluated quality of work was 7.92. The mean values by the different occupational for the quality of work in their respective work units were as follows: physicians 8.26, research staff 7.97, maintenance staff 7.81, office and administrative staff 8.10 and nursing staff 7.84 (Table 1).

**Table 1:** **Demographics of the hospital staff respondents** (**N** = **1424**) (**n**, %)

**Background variable**	**Nursing staff (n** **=** **779), %**	**Physicians (n** **=** **147), %**	**Research staff (n** **=** **65) %**	**Maintenance staff (n** **= 237), %**	**Office and administrative staff (n** **=** **196), %**
Gender					
Female	88	61	89	86	92
Male	12	39	11	14	8
Age					
≤30 years	13	1	8	8	8
31–40 years	24	26	20	15	11
41–50 years	31	41	44	35	35
≥51 years	32	32	28	42	46
Work experience in current unit					
≤1 year	14	18	11	14	11
2–5 years	28	23	28	34	30
6–10 years	19	23	14	20	16
11–20 years	20	26	31	13	22
≥21 years	19	10	16	19	21
Overall work experience					
≤1 year	3	1	2	4	2
2–5 years	10	6	9	15	11
6–10 years	17	14	13	17	7
11–20 years	26	36	36	18	22
≥ 21 years	44	43	40	46	58
Type of employment					
Permanent	82	72	75	79	86
Temporary	18	28	25	21	14
Working hours					
Day	38	99	100	36	95
Rotational	62	1	0	64	5
Quality of work					
4–6	8	5	5	10	4
7–8	72	50	67	69	64
9–10	20	45	28	21	32

### Job satisfaction evaluations

Overall, the university hospital staff respondents rated their job satisfaction as good (M = 3.74, SD = 0.57). The highest value was given for the subarea of motivating factors of the work (M = 4.27, SD = 0.60). Working welfare (M = 4.19, SD = 0.58), leadership (M = 3.82, SD = 0.90) and sense of community (M = 3.72, SD = 0.81) were also fairly strong subareas of job satisfaction. Requiring factors of work (like enough staff, appropriate work load and salary) (M = 3.25, SD = 0.77), working environment (M = 3.47, SD = 0.94) and participation in decision-making (M = 3.49, SD = 0.84) were considered to be the worst subareas (Table 2).

**Table 2:** **Job satisfaction scores of hospital staff** (**Mean**, **SD**, ***P***-**value**)

**Staff group**	**Leadership**	**Requiring factors of the work**	**Motivating factors of the work**	**Working environment**	**Working welfare**	**Participation in decision-making**	**Sense of community**	**Job satisfaction**
	**Mean (SD)**	**Mean (SD)**	**Mean (SD)**	**Mean (SD)**	**Mean (SD)**	**Mean (SD)**	**Mean (SD)**	**Mean (SD)**
Nursing staff (n = 779)	3.86 (0.91)	3.16 (0.76)	4.27 (0.60)	3.28 (0.95)	4.19 (0.57)	3.44 (0.84)	3.76 (0.80)	3.71 (0.56)
Physicians (n = 147)	3.87 (0.83)	3.27 (0.87)	4.38 (0.52)	3.76 (0.80)	4.07 (0.65)	3.76 (0.77)	3.70 (0.87)	3.81 (0.61)
Research staff (n = 65)	3.84 (0.67)	3.36 (0.64)	4.28 (0.61)	3.57 (0.92)	4.16 (0.50)	3.48 (0.82)	3.78 (0.65)	3.78 (0.48)
Maintenance staff (n = 237)	3.62 (0.97)	3.23 (0.76)	4.25 (0.60)	3.53 (0.91)	4.22 (0.56)	3.48 (0.85)	3.52 (0.87)	3.69 (0.59)
Office and administrative staff (n = 196)	3.85 (0.87)	3.58 (0.67)	4.21 (0.66)	3.93 (0.84)	4.25 (0.61)	3.53 (0.88)	3.81 (0.72)	3.88 (0.54)
All staff groups (n = 1424)	3.82 (0.90)	3.25 (0.77)	4.27 (0.60)	3.47 (0.94)	4.19 (0.58)	3.49 (0.84)	3.72 (0.81)	3.74 (0.57)
*P*-value	0.008	< 0.0001	0.255	< 0.0001	0.065	< 0.001	0.004	< 0.001

(Scores 1–5; 1 = lowest, 5 = highest, *P* < 0.05).

### Differences in job satisfaction between the staff groups

The physicians were more satisfied (M = 3.87, SD = 0.83) with leadership than the other staff groups (M = 3.62–3.86), while the maintenance staff were least satisfied (M = 3.62, SD = 0.97). The staff groups’ evaluations of leadership (*P* = 0.008) and the requiring factors differed from each other (*P* < 0.0001). The nursing staff gave lower scores for the requiring factors of work (M = 3.16, SD = 0.76) than the other groups (M = 3.27–3.58). The office and administrative staff group was the most satisfied with requiring factors (M = 3.58, SD = 0.67). The mean scores for motivating factors of the work ranged from 4.21 (office and administrative staff) to 4.38 (physicians). Differences were detected between the different personnel groups’ evaluations of the working environment (*P* <0.0001). The mean score was highest (M = 3.93, SD = 0.84) among the office and administrative staff, and lowest (M = 3.28, SD = 0.95) among the nursing staff. Working welfare was scored highest by the office and administrative staff (M = 4.25 SD = 0.61) and lowest by the physicians (M = 4.07, SD = 0.65). The personnel groups’ evaluations of participation in decision-making also differed significantly (*P* < 0.001). It was highest among the physicians (M = 3.76, SD = 0.77) and lowest among the nursing staff (M = 3.44, SD = 0.84). There were also differences in the perceived sense of community between the staff groups (*P* = 0.004). The office and administrative staff’s evaluation was highest (M = 3.82, SD = 0.72), whereas the maintenance staff’s evaluation was the lowest (M = 3.52, SD = 0.87). Mean scores of overall satisfaction also differed between the staff groups (*P* <0.001), being highest among the office and administrative staff (M = 3.88, SD = 0.54) and lowest among the maintenance staff (M = 3.69, SD = 0.59) (Table 2).

### Relationship between quality of work and job satisfaction

Staff’s evaluations of the quality of work in their respective working units had a clear relationship with experiences of job satisfaction. Those who evaluated the quality of work as excellent also rated their job satisfaction to be the highest in all subscales (*P* < 0.0001).

## Discussion

The objectives of this study were to evaluate job satisfaction of all staff working at a Finnish university hospital, identify the differences in job satisfaction between staff groups and explore the relationship between their self-evaluated quality of work and job satisfaction.

Overall, the results indicate that the staff of the university hospital were satisfied with their jobs, in agreement with previous findings of generally high job satisfaction among staff in numerous hospitals [4-7,9-11,20,23-27]. Working at the university hospital was viewed as strongly motivating by all staff groups, which can be regarded as essential for high job satisfaction. However, satisfaction with the requiring factors of work, working environment and participation in decision-making was scored the lowest by nursing staff. The nurses felt that the number of the staff was sometimes too low. The time available for each patient was often inadequate, and therefore the work was sometimes too demanding and stressful. The hospital buildings were also considered partly cramped, which sometimes made working uncomfortable. The hospital administration was also seen as bureaucratic, and the staff did not feel they had many opportunities to participate in decision-making [4]. Another relatively dissatisfied staff group was the maintenance staff. The most satisfied staff groups were the office and administrative staff, and physicians. Aasland *et al*. [7] and Szecsenyi *et al*. [11] have also reported that physicians (in Norway and Germany, respectively) were highly satisfied with their jobs, and several previous studies e.g. [5], have shown that physicians were more satisfied with their jobs than nurses. In contrast, in an earlier analysis of nursing staffs’, physicians’ and leaders’ views of organisational factors (which partly correspond to the variables used to measure job satisfaction in the present study), Kvist *et al*. [29] found evidence that physicians were most dissatisfied with their jobs. However, since then there have been numerous improvements in working conditions and salaries of Finnish physicians. On the other hand, leaders evaluated their job satisfaction most highly [29], a finding supported by the present study, as they were included in the office and administrative staff group. The staff groups that evaluated the quality of work to be excellent were also the most satisfied, in agreement with our previous study [23].

Our results also indicate that the KUHJSS is a reliable instrument for evaluating the job satisfaction of all the staff working in a hospital. It clearly showed that all staff groups identified the same strong and weak areas of job satisfaction, suggesting that they all understood and evaluated job satisfaction in a similar manner, as Etchegaray et al. [8] reported.

### Limitations

The response rate (33%) of the present study was moderate but lower than for our study conducted in 2008 (47%, both web based and mailed methods) [23] and much more lower than in our earlier study [29], in which the rate was 63% after reminders. The response rate was substantially lower than expected considering that this was the first time that data have been collected electronically from all staff. The staff receive numerous research surveys, and therefore their motivation to respond to them may have been low. We need to be cautious in generalising the results for all staff at the university hospital. However, the results give an indication of the overall job satisfaction in the study hospital. It was impossible to draw any conclusions about the staff that did not respond, because the survey was conducted anonymously.

## Conclusions

This study shows that the work of physicians and nursing staff at a university hospital is regarded as challenging and motivating, which thus provides a good foundation for job satisfaction. Nursing staff were found to be the least satisfied group at the university hospital, primarily because they considered their work to be too demanding. Nursing leaders should thus critically examine staffing levels and caring models in different units. Working welfare is a key element of job satisfaction. Therefore, staff welfare at work should be promoted by implementing operational models developed jointly by employers, managers and representatives of occupational healthcare representatives. A university hospital is a complex, continuously changing and highly challenging work environment. All hospital managers should continuously evaluate information obtained from job satisfaction evaluations. Each staff member has an influence on job satisfaction in his/her work unit. There is a need to develop a culture of participation in the hospitals to foster a sense of appreciation among staff.

## Abbreviations

KUHJSS: Kuopio University hospital job satisfaction scale.

## Competing interests

The authors declare that they have no competing interests.

## Authors’ contributions

TK, RM and KV-J contributed to the design of the study. TK and RM collected, analyzed and interpreted the data and results. TK, RM and KV-J were involved in drafting and critically revising the manuscript, as well as reading and approving the final manuscript.

## Pre-publication history

The pre-publication history for this paper can be accessed here:

http://www.biomedcentral.com/1472-6963/13/376/prepub

## Supplementary Material

Additional file 1 Kuopio University Hospital Job Satisfaction Scale (KUHJSS). Click here for file
