# Supplementary material for: Does Finnish hospital staff job satisfaction vary across occupational groups?
Source: BMC Health Serv Res. 2013 Oct 2;13:376. doi: 10.1186/1472-6963-13-376 (PMC3852482; doi:10.1186/1472-6963-13-376)
Supplement: Additional file 1 — Kuopio University Hospital Job Satisfaction Scale (KUHJSS). [file 1472-6963-13-376-S1.doc]

**STAFF JOB SATISFACTION SCALE**

Background information:

1. Gender 1. female

2. male

2. Age ____ years

3. Unit _________

4. Division _________

5. Position 1. Physicians

2. Research staff

3. Maintenance staff

4. Office and administrative staff 5. Nursing staff

6. Work experience in current unit: ____ years

7. Overall work experience : _____ years

8. Type of employment: 1. permanent position

2. non-permanent/temporary position

9. Working hours: 1. daytime work

2. period/shift work

10. How is the quality of work in your unit? _________ (4-10; 4=worst, 10=best)

Please answer the following statements by choosing the alternative which best describes your work, work unit, working environment or organization.

Fully Partly Cannot Partly Fully

disagree disagree say agree agree

11. My manager/director is genuinely

interested in the well-being of the staff. 1 2 3 4 5

12. My manager/director treats the staff

fairly and equally. 1 2 3 4 5

13. My manager/director provides the staff

feedback with the aim of developing work. 1 2 3 4 5

14. My manager/director informs me thoroughly

about issues concerning my unit. 1 2 3 4 5

15. My manager/director enables the

continuous professional development

of the staff. 1 2 3 4 5

16. My manager/director encourages staff

to take part in the planning of our unit’s

operation. 1 2 3 4 5

17. My manager/director is interested in

work results and outcomes. 1 2 3 4 5

18. My work unit is safe and secure. 1 2 3 4 5

19. My work unit is comfortable. 1 2 3 4 5

20. The workload is distributed evenly

in my unit. 1 2 3 4 5

21. I am satisfied with my working hours. 1 2 3 4 5

22. There are usually enough staff in my unit. 1 2 3 4 5

23. New employees are welcomed

in my unit. 1 2 3 4 5

24. I trust the expertise of my colleagues. 1 2 3 4 5

25. There is a good community spirit in my unit. 1 2 3 4 5

26. The flow of information works well

in my unit. 1 2 3 4 5

27. My work load is appropriate. 1 2 3 4 5

Fully Partly Cannot Partly Fully

disagree disagree say agree agree

28. I have opportunities to plan my work

independently. 1 2 3 4 5

29. I have opportunities to make independent

decisions in my work. 1 2 3 4 5

30. My work tasks are suitably challenging. 1 2 3 4 5

31. I can apply a wide range of my skills

and expertise in my work. 1 2 3 4 5

32. Client feedback motivates me in my work. 1 2 3 4 5

33. My work is interesting. 1 2 3 4 5

34. The upper management of the hospital

district appreciates my work. 1 2 3 4 5

35. My unit has appropriate equipment

to ensure quality of care. 1 2 3 4 5

36. My salary is appropriate in relation to the

demands of my work. 1 2 3 4 5

37. I have a chance to influence

decision-making in my unit. 1 2 3 4 5

38. My unit has appropriate work

facilities. 1 2 3 4 5

39. I have a chance of career development

in the hospital. 1 2 3 4 5

40. I am willing to work in this hospital district

in the future. 1 2 3 4 5

41. I appreciate my own work. 1 2 3 4 5

42. Combining work and personal life is

successful. 1 2 3 4 5

43. I am active in developing myself

professionally. 1 2 3 4 5

44. I do not find my work too stressful. 1 2 3 4 5

45. I feel I am a competent employee. 1 2 3 4 5

46. I look after my personal well-being. 1 2 3 4 5

47. I am happy with my current health. 1 2 3 4 5

THANK YOU FOR YOUR RESPONSES!
